# Integrating a New Online Platform in Primary Care for Early Detection, Referral, and Intervention in Autism Spectrum Disorder: The First Italian Pivotal Clinical Study

**DOI:** 10.3390/brainsci12020256

**Published:** 2022-02-12

**Authors:** Paola Colombo, Noemi Buo, Silvia Busti Ceccarelli, Massimo Molteni

**Affiliations:** Child Psychopathology Unit, Scientific Institute, IRCCS Eugenio Medea, Bosisio Parini, 23842 Lecco, Italy; noemi.buo@lanostrafamiglia.it (N.B.); silvia.busti@lanostrafamiglia.it (S.B.C.); massimo.molteni@lanostrafamiglia.it (M.M.)

**Keywords:** autism spectrum disorder, online screening, early detection, primary care, screening CHAT

## Abstract

Autism spectrum disorder (ASD) is a heterogeneous condition characterized by deficits in social communication and a repetitive pattern of behavior, with recent Italian prevalence estimates of 1 in 77. Although the core behavioral features of ASD appear to emerge within the first two years of life, clinical diagnosis is often not received before the third birthday. The American Academy of Pediatrics (AAP) has recommended that primary care physicians routinely screen for ASD at the 18- and 24-month visits. In Italy, the Guidelines of the Italian National Institute for Health (ISS) recommend the Checklist for Autism in Toddlers (CHAT) as a screening tool for ASD, which compares parent responses with a semistructured observation by a healthcare provider. In the Italian National Health System, pediatricians have regular visits with all children; however, there is wide variability in following screening guidelines, and some barriers have been detected. For these reasons, other studies have highlighted the advantages of using telemedicine with the potential for enhancing ASD screening practices. The current study is an examination of the implementation of the first Italian online web-based screening tool (Web Italian Network for Autism Spectrum DisorderWIN4ASD), an innovative web app for pediatricians. We present the data obtained from the screening activity through the platform by a small group of pediatricians. The results of this study show that the implemented web-based platform appears to be an effective, efficient, and sustainable way to integrate screening services into primary care.

## 1. Introduction

Autism spectrum disorder (ASD) is a heterogeneous condition characterized by deficits in social communication and a repetitive pattern of behavior [1]. There has been an unprecedented increase in the prevalence of ASD among children in recent decades, with recent Italian prevalence estimates of 1 in 77 in the range of seven to nine years old [2].

The core behavioral features of ASD appear to emerge within the first two years of life, and experienced professionals can make a diagnosis of ASD in the second year of life as an essential step for early intervention. Studies have shown that early interventions provide effective benefits in improving children’s developmental trajectory and are often associated with more positive outcomes in communication, social interaction, and cognitive development [3].

Despite the fact that scientific knowledge about early identification is continuously increasing, the delay between parents’ first concerns regarding the atypical development of their child and receiving a diagnosis of ASD is still long, and subsequent access to early intervention may add up to four years. Even though the age at first diagnosis has gradually decreased during the last decade, population-based studies have revealed that clinical diagnosis is often not received before the age of three years [4,5,6] and is further delayed for children from underserved areas and low-income and minority families [7]. Barriers to earlier diagnosis include the gradual onset and heterogeneity of ASD symptoms, as well as the lack of a standardized process to ensure that children are systematically monitored for early clinical markers of ASD to promote an earlier diagnosis.

The widespread diffusion of active surveillance systems, which includes the use of standardized and specific screening tools for neurodevelopmental disorders, would allow for timely and early assessments, with strong advantages for the well-being of children and their families.

The American Academy of Pediatrics (AAP) has recommended that primary care physicians caring for toddlers routinely screen for ASD at the 18- and 24-month visits [8,9]. However, AAP surveys of pediatricians have suggested wide variability in following the screening guidelines. Although the rate of pediatricians reporting the use of a standardized tool for developmental screening increased from 21 to 63% from 2002 to 2016 [10], there are several potential barriers to ASD-specific screening within the healthcare system. Several obstacles to screening have been reported by pediatricians: a lack of time and inadequate reimbursement, a lack of familiarity with tools, difficulty with scoring, and a lack of direct and effective referral systems and monitoring of outcomes [9]. In general, GPs report low comfort with assessments of ASD risk [11], and while the increase in specific training programs on neurodevelopmental disorders addressed to GPs has an impact on improving clinician knowledge, they provide little support in achieving practice change [12]. These hindrances could prevent appropriate and timely care for children with ASD.

Digital screening potentially offers an answer to the logistical challenges of implementing a surveillance system based on administering a screening tool. Studies seeking to improve ASD screening systems have shown that the introduction of an electronic form and digital decision support can significantly impact the screening process and aid the development of a healthcare network between pediatricians and specialist services [13,14].

Based on these premises, over the past few years, Scientific Institute E Medea—Associazione La Nostra Famiglia (LC) has been working on developing and implementing tele-mental health systems for the early screening of autism spectrum disorder in agreement with local health authorities.

The current study examined the implementation of the first Italian online web-based CHAT screening tool (Web Italian Network for Autism Spectrum Disorder—WIN4ASD) for pediatricians in a systematic autism screening process for all toddlers at their 18-month well-child pediatric visits as part of an active surveillance program for neurodevelopmental disorders in the first 36 months of age.

We provide data derived from this pivotal clinical study based on the IT platform activity.

The principal aim of this work, which represents the most significant challenge, was to present an innovative digital tool meant for building a network between the primary care system and specialist services in order to facilitate the early identification and diagnosis of ASD children among the general population. The Italian Public Health System involves family pediatricians to accomplish healthcare activities, health promotion, and active surveillance actions on neurodevelopmental disorders for each assigned patient aged 0–16 years old. In particular, these activities are carried out during health-check-up visits at well-defined development time points, one of which is at 18 months of age.

Furthermore, the Italian Public Health System includes neuropsychiatric services for children and adolescents, which include the diagnosis, treatment, and rehabilitation of neurodevelopmental disorders and other neurological and psychiatric developmental diseases. These services have a wider catchment area and are not homogeneously distributed throughout the country; they are well-represented in some Italian regions, particularly in Lombardy.

This work is aimed at introducing the Win4ASD platform, an innovative web-based app that has been developed by a multi-disciplinary team of researchers, clinicians, and informatics professionals, and includes an ASD screening tool that can be used at 18-month well-child visits.

The platform allows us to provide data and discuss three interconnected topics:

1. The empowerment of the network:

The web app is designed to simplify and facilitate communication between GPs and child neuropsychiatric public services. A measure of the strength of the network created is the number of pediatricians and specialists using the app.

2. The construction of a surveillance system:

The Win4asd platform was designed to improve the ability of pediatricians to identify early children at clinical risk of ASD in the general population by recognizing early clinical markers; to screen a population of children aged 18–20 months through the administration of CHAT screening tool (Checklist for Autism in Toddlers) [15]; to achieve a reduction in the average age of the first diagnosis of ASD.

3. The feasibility study:

Using performance measures of the platform’s activities, this study aims to investigate the potential of telemedicine in the field of neurodevelopmental disorders, specifically Autism Spectrum Disorders.

## 2. Materials and Methods

### 2.1. Screening Tool

Surveillance for autism spectrum disorder is recommended by the Guidelines of the National Institute for Health (ISS), and CHAT questionnaire [15] is one of the screening tools recommended on the Guideline of the Italian Society of Childhood and Adolescence Neuropsychiatry (SINPIA), the most important scientific association in the field of prevention, diagnosis, treatment, and rehabilitation of neurological, neuropsychological, and psychiatric disorders of childhood and adolescence.

The Italian translation of the screening tool is available on the Autism Research Centre website [16].

The Checklist for Autism in Toddlers is a screening instrument aimed at detecting children aged 18 months who are at risk of autism. It was validated in England on a population of 16,000 children and was designed to be administered by primary healthcare services. The checklist is composed of two sections: the nine questions in Section A are asked of the parents by the GPs, who then observe the child’s behavior directly and complete the five items in Section B. All items are rated as “yes” or “no.”

This standardized screening tool is available to GPs through the web app. It takes 5–10 min to administer, and requires the pediatrician’s direct observation and involvement in the entire process, thereby increasing their compliance.

### 2.2. Procedure

An ad hoc web platform (www.win4asd.it, accessed on 30 December 2021) was designed using LAMP technology in order to easily manage the entire screening process.

A group of pediatricians was invited to voluntarily participate in the pivotal clinical study. Dedicated training on the early signs of autism spectrum disorder and on information and communications technology tools was administered to the pediatricians involved in the study. The timely recognition of the clinical markers of ASD can lead to earlier diagnosis and treatment, as well as the best potential outcomes. After the training, the screening activity started.

The pediatricians administered the CHAT screening through the WIN4ASD app during 18-month well-child visits, which are mandatory for all children in the Italian Health System (SSN). After finishing the test, the web app performs automatic scoring and guides the GP through different choices based on the result.

The screening result can be: high risk (failure of all critical items); medium risk (failure of a few critical items); or generic risk—a risk of other neurodevelopmental disorders (failure of a few non-critical items).

When the result is “high risk,” the GP informs the parents and suggests that the child be referred to one of the networked specialist healthcare centers for additional extensive diagnostic evaluation and subsequent appropriate intervention. The referral function is automatically activated; the pediatrician can select a preferred service or provider and can then send the screening output via a dedicated form in the web app. In this case, the child was uniquely identified by her/his fiscal code (FC), an alphanumeric code made up of 16 characters that is issued by the public authority to any Italian citizen and used to access the public health system. Patients requiring an immediate diagnostic evaluation are processed in the first few days (Fast-Track) by the chosen specialist service.

When the result is “medium/generic risk”, short-term monitoring is required. The GP should schedule an appointment for one month later for further testing of the child (re-administration of the CHAT screening); a reminder is automatically sent to the pediatrician by email. When the result is “no risk,” there is no recommended action. The results are simply anonymously recorded in the database for statistical purposes.

We built a secure interconnection via the web app, which facilitates the exchange of clinical information between the GP and the child psychiatry specialist for when a patient is referred to a service. They can share the performed screening test and any other clinical information useful to facilitate the diagnostic process and subsequent clinical care, including spontaneous short films recorded by the parents.

## 3. Results

### 3.1. The Web Italian Network for Autism Spectrum Disorder—The WIN4ASD Platform

This work began alongside a research project, “Italian Autism Spectrum Disorders Network: Filling the Gaps in the National Health System”, which allowed the design and first implementation of the web-based platform, available at www.win4asd.it, accessed on 30 December 2021.

The web app was designed using LAMP open-source technology (Linux, Apache, PHP, and MySQL) and responsive technology (HTML 5, CSS3, and jQuery). It can be used on any device (PC, tablet, and smartphone), it is extremely user-friendly and accessible, and fully conforms to the safety requirements (the app is hosted in GARR servers, the ultra-broadband network dedicated to the Italian research and education community). The web platform manages sensitive data, both anonymously and personally, using the HTTPS protocol for greater security; two-stage access is required by receiving a temporary code by SMS after entering the username and password.

**Figure 1:**
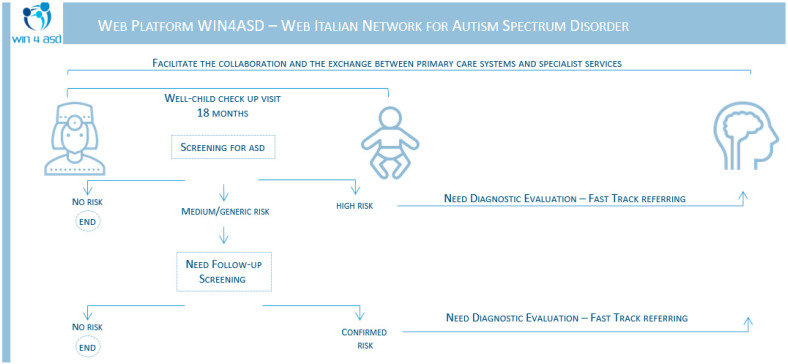
The WIN4ASD web app workflow.

Different modules are available on the web platform. The CHAT questionnaire administration module involves a few simple actions: input of patient personal data, screening-test administration, and screening-test saving and printing. A screening-scoring algorithm was integrated into the web platform, which can generate an automatic and immediate individual patient output (Figure 1).

### 3.2. The Empowerment of the Network

In this pivotal clinical study, conducted from January 2018 to November 2019, 20 pediatricians were involved. Out of the 18 logged into the platform, 15 were active. In order to understand the pediatricians’ involvement, the average number of CHAT questionnaires administered by each GP was calculated. In the first phase, the average number of screenings administered by each pediatrician was 29 and ranged from a minimum of three to a maximum of 70 screenings per pediatrician.

A second group of 21 pediatricians from another district was subsequently voluntarily involved. They had access to the platform starting from October 2019, with an average number of CHATs/pediatricians of 66 (min. = 3; max. = 94).

As a result of the progressive extension of the web platform to an increasing number of pediatricians starting in May 2020, the web app has gradually been distributed and extended to a wide group of family pediatricians working concurrently with the agreements with regional health organizations in the Lombardy region.

After the health emergency caused by COVID-19, an online training program was distributed freely through the web app, which was already being used by a large number of pediatricians.

The platform currently has a total number of 451 registered and logged-in family pediatricians.

### 3.3. The Construction of a Surveillance System: Screening Outcome and Fast-Track Referral

Data on screening activity were gathered in the period of January 2018 to November 2021. The screening activity continued despite the strong slowdown due to the COVID-19 emergency.

Overall, the screening CHAT questionnaire was administered to 1250 patients in the general population (50.1% males) during the 18-month well-child visits. A total of 1178 children (94.24%) matched the “no risk” criteria and exited the screening process, whereas 72 children (5.76%) matched a risk profile: 36 (2.88%) were at “generic risk”, 21 (1.69%) were at “medium risk”, and 15 were at “high risk” (1.20%). One month later, all of the medium- and generic-risk groups were re-screened, as required by the protocol.

Twenty-six patients were referred to specialist services, and the Fast-Track diagnostic process was performed by the autism clinical team.

**Figure 2:**
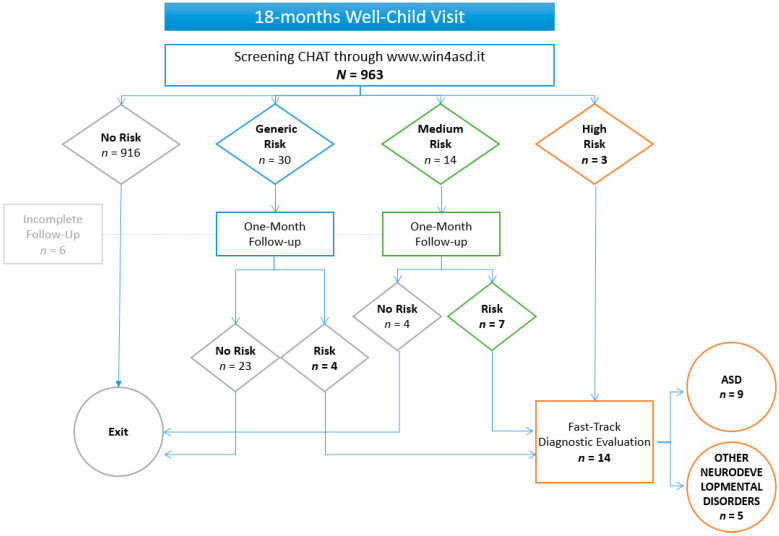
Flowchart showing screening results and diagnostic outcomes.

Data on diagnostic outcomes are available only for the screening activity performed by 36 GPs, who carried out a total of 963 screenings. As shown in Figure 2, on the 963 screened children, the first screen was negative (no risk) in 95.12% (*N* = 916), whereas 47 children matched a risk profile—30 (3.11%) had “generic risk,” 14 (1.45%) had “medium risk,” and three had “high risk” (0.31%). A total of 38 children (3.95%) were screened twice, and 11 confirmed the risk. All of the 14 children invited for diagnostic evaluation completed the diagnostic process. ASD diagnoses were confirmed for nine patients, and five patients received a diagnose of another neurodevelopmental disorder. The child neuropsychiatry services subsequently handled the care management of all referred patients.

### 3.4. The Feasibility Study

To evaluate the quality of the system and to describe it with a quantitative measure that can be replicated and compared over time, we adopted the indicators recommended by the Italian National Guidelines for telehealth [17]. The performance measures available for the data collected between January 2018 and November 2021 are provided in Table 1. This process is a work in progress, and in the future we aim to be able to capture more indicators.

**Figure 3:**
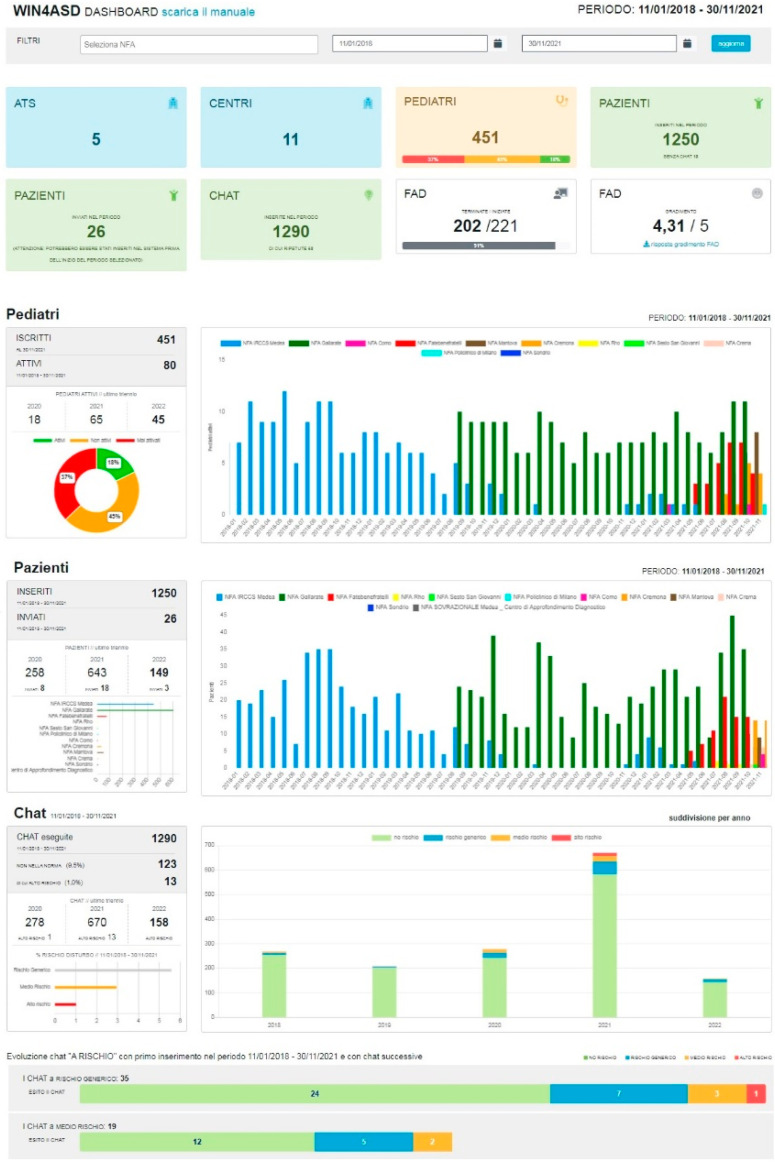
WIN4ASD dashboard.

Furthermore, a visual representation of performance, with charts and graphs, was developed in an interactive dashboard (Figure 3) to provide information at a glance. The dashboard receives information from a linked database and carries out descriptive statistics with group-level output. It shows data on pediatricians’ activity over time, the number of patients over time, the number of screening tests administered, and the outcomes of the screening follow-up, and can be queried by selecting different criteria and can display different information.

The tool is available to the Pivot Centre project manager as well as to the Public Health Authorities that are involved. It allows to monitor performance, identify trends, generate detailed reports, and set estimates and targets to make more informed decisions.

## 4. Discussion

In this work, we presented an innovative digital tool aimed at facilitating the early identification and diagnosis of ASD children among the general population and encouraging GPs and child neuropsychiatry specialists to connect in a health network. The principal aim of this work represents the most significant challenge: to improve the health system to enhance the continuity of care in the field of neurodevelopmental disorders, specifically for Autism Spectrum Disorder, by employing digital e-health tools. Within this challenge, the quality of communication between GPs and specialists seems to be a crucial aspect, which improves the results of treatment and patient satisfaction [18]. Advances in communication technology have increased the potential methods and speed by which healthcare professionals can communicate, permitting the easy and instantaneous transfer of patient information between GPs and specialists. To respond to this challenge, a very simple and innovative web-based telemedicine system was proposed, aimed at developing an effective health network to deal with chronic conditions such as autism.

The empowerment of the network: We described the WIN4ASD web app’s architecture and features. It is a simple and useful tool that proposes a surveillance system model for the early detection of ASD risk, that can be easily spread and scaled. In the past few months, the WIN4ASD web app has progressively been made available to primary-care pediatricians in the Lombardy region, as well as to related special services. Currently, it has over 450 registered pediatricians and exponential growth.

We presented data from the pivot study, which looks promising. The supported network between GPs and specialist services proves the effectiveness of the proposed model. The results of this study show that the implemented web-based platform appears to be an effective, efficient, and sustainable way to integrate screening services into primary care.

The construction of a surveillance system: Our results on screening activity, although to be observed as the results of a pivot study, confirm the high specificity of the CHAT; all 14 referred patients received a diagnosis of autism spectrum disorder (most cases) or another neurodevelopmental disorder. Furthermore, low sensitivity is not a clinical problem in a public health system such as Italy’s.

Moreover, once a child has been identified as being at risk of ASD through screening, a direct referral to a specialist service speeds up the diagnostic process, reduces the time between the first concerns and the diagnosis, and improves family compliance with the evaluation process [19].

The feasibility Study: Finally, the web app’s performance measures show an ongoing and expanding active service. Through a simple and safe digital tool, we created a health network where the collaboration between specialist services and GPs can also facilitate the care of ASD children in a public health system.

The COVID-19 pandemic has increased the use of telemedicine as a way to address the need for diagnostic clarification in young children at risk of autism spectrum disorder (ASD). The uncertainty that has emerged during the COVID-19 emergency requires rethinking the models of innovation and improvement in the health system, especially toward digital transformation. It has brought the opportunity to set up effective and more widespread tools, representing a possible, robust, and appropriate solution in this emergency.

This study has some limitations. First, although we proposed a systematic screening tool for the general population, this was not an epidemiological study. This is due to a small sample of involved GPs, and most importantly, to their voluntary participation in the research process. Thus, the data on screening outcomes cannot have epidemiological significance, even though our rate of high-risk subjects is in line with the findings in the literature and confirms the potentiality of the proposed screening system to identify subjects with ASD risk. Second, we do not currently have complete data on diagnostic outcomes, since the web app does not yet provide this information. Finally, the web app does not collect direct information on user experience; a customer satisfaction questionnaire must be implemented.

## 5. Conclusions

In this work, we presented an innovative digital tool aimed at facilitating the ear-ly identification and diagnosis of ASD children among the general population and en-couraging GPs and child neuropsychiatry specialists to connect in a health network. Even though there is a consensus within the scientific community that early in-tervention based on early detection is important, implementing this strategy and es-tablishing links among different stakeholders in this field remains challenging. This work presents a feasible model for improving the health system to enhance the conti-nuity of care in the field of neurodevelopmental disorders, specifically for Autism Spectrum Disorder, by employing digital e-health tools.

## Figures and Tables

**Table 1:** Performance measures of the WIN4ASD web app (* data on 2021 refers to January–November).

	Measure	2018	2019	2020	2021 *
**Size**	Absolute (no. of users followed/12 months)	268	211	258	514
	Average (average number of contacts/month)	22.33	17.58	21.50	42.36
**Continuity**	Duration of service	12 months	12 months	12 months	12 months
	Standard deviation (no. of patients/month)	8.83 (min.: 7; max.: 35)	8.71 (min.: 4; max.: 36)	21.50 (min.: 9; max.: 38)	24.96 (min.: 22; max.: 100)

## Data Availability

The data that support the findings of this study are available from the corresponding author, P.C., upon reasonable request.
